# Antibacterial Effects of Various Types of Bee Products in Malaysia: A Systematic Review

**DOI:** 10.21315/mjms2024.31.3.3

**Published:** 2024-06-27

**Authors:** Nur Aliah Mohamad, Alaa’ Fahed Al-Emerieen, Ahmad Adebayo Irekeola, Rafidah Hanim Shueb

**Affiliations:** 1 Department of Medical Microbiology and Parasitology, School of Medical Sciences, Universiti Sains Malaysia, Kelantan, Malaysia; 2 Department of Biological Sciences, Microbiology Unit, College of Natural and Applied Sciences, Summit University Offa, Kwara State, Nigeria; 3 Institute for Research in Molecular Medicine (INFORMM), Universiti Sains Malaysia, Kelantan, Malaysia

**Keywords:** honey, bee products, Malaysia, antibacterial, antimicrobial

## Abstract

Antibiotics are widely used to treat bacterial infections. The effectiveness of antibiotics is very important, but unfortunately, prolonged exposure leads to the development of antibiotic resistance in some bacteria. Hence, using natural products as antibacterial agents is an attractive alternative, given that they have been used as traditional medicine since the existence of humanity. This study systematically reviewed the antibacterial activity of Malaysian bee products such as honey, propolis and bee bread. Five electronic databases: i) PubMed; ii) ScienceDirect; iii) Scopus; iv) Web of Science Core Collection and v) Google Scholar, were searched for relevant articles. A total of 153 articles were obtained from the initial search. Of these, 32 articles, including 24 on honey, eight on propolis and one on bee bread, were selected based on inclusion and exclusion criteria. Most studies reported that honey, propolis and bee bread demonstrated antibacterial properties against Gram-positive and Gram-negative bacteria.

## Introduction

Bee products are natural products utilised in traditional medicine because of the abundant presence of bioactive compounds within these products, as highlighted by their rich content of bioactive molecules and advantageous health attributes that encompass potent healing properties. Bee products include honey, propolis, royal jelly, bee wax, bee venom and bee bread (1). Honey is a liquid, ranging in colour from light to dark amber, made by honey bees from floral nectar or honeydew. The bees transport the nectar of flowers or honeydew into the hives, where they start the processes that eventually turn them into honey (2). Propolis, also known as ‘bee glue’, is a sticky substance produced from a mixture of bee wax with resinous sap obtained from tree buds or other flowering plants (3). Royal jelly is generated directly by bees using secretions from their hypopharyngeal and mandibular salivary glands, specifically from young worker bees. It serves as nourishment for both the queen bee and the larvae, although its use for the latter is limited to just a few days (4). In contrast, bee wax is obtained by mixing the pollen oils with white wax to form a brown or yellow colour, and bee venom is an acidic colourless liquid secreted by the venom gland located in the abdominal cavity of the bees, while bee bread is a mixture of pollen and flower nectar or honey (5).

These bee products are becoming reasonably significant and are also known to contain several great bioactivities. In folk medicine, society used propolis to support a healthy life, royal jelly to support the body’s defence mechanism and boost energy, honey to treat burns and sore throats and a natural sweetener in cooking (6). There has been extensive research examining the health benefits of bee products. Anti-inflammatory, antifungal, antibacterial, antioxidant and antiviral effects have all been linked to valuable health effects of bee products. In addition, honeybee products also demonstrate antitumour properties (7). Carbohydrates, proteins, peptides, lipids, minerals, vitamins, polyphenols, flavonoids, terpenoids and a trace amount of other compounds are among the biologically active elements of bee products that contribute to their great bioactivities (1).

Bacteria can cause infections in different parts of the human body (8). For example, *Staphylococcus aureus* and *Streptococcus pyogenes* cause wound infection, *Escherichia coli* causes gastrointestinal infections (9) and *Streptococcus mutans* is responsible for dental caries and biofilm formation on teeth (10). Prolonged exposure to or misuse of antibiotics leads to antibiotic resistance in bacteria such as methicillin-resistant *Staphylococcus aureus* (MRSA) and carbapenem-resistant *Klebsiella pneumonia*. Thus, it is essential to discover alternative antibacterial agents that do not require antibiotics (8). A systematic review was conducted to study the antibacterial effects of bee products in Malaysia.

## Method

Literature analysis was conducted while adhering to the guidelines outlined in the Preferred Reporting Items for Systematic Reviews and Meta-Analyses (PRISMA).

### Literature Search

Five electronic databases (PubMed, Scopus, ScienceDirect, Google Scholar and Web of Science Core Collection) were searched and the keywords used were as follows: Honey OR ‘royal jelly’ OR propolis OR ‘bee wax’ OR ‘bee venom’ OR ‘bee bread’ AND Malaysia OR Malaysian AND antibacterial OR antimicrobial. The detailed search strategy is provided in the Appendix file. The first search was conducted on 25 October 2022, the updated and last search was done on 25 February 2023 and 153 references were found. The references were exported to Mendeley’s desktop and duplicates were removed.

### Inclusion and Exclusion Criteria for Studies

All the full-length in vitro studies and research articles related to the antibacterial and antimicrobial activity of any bee products from Malaysia were considered for inclusion. We excluded data on studies other than antibacterial and antimicrobial activities, studies using bee products from other countries, reviews, articles whose full texts cannot be accessed and any unrelated results. Studies on the combination or synergistic effect of bee products with other natural products were also excluded. The records found were screened by two writers (NAM and AFAE) based on determined inclusion and exclusion, and those irrelevant by titles and abstracts were removed. The articles that met the requirements for inclusion were then subjected to full-text screening.

### Data Extraction and Analysis

The extraction of data was done based on the objective. Data such as the name of authors and year published, types of bee products used, bacteria tested, methodological aspects, outcomes, or results were extracted in Microsoft Word by two authors (NAM and AFAE). A consensus with an additional author (AAI) resolved disagreements among the authors. Citation of authors and year published was done using Mendeley desktop.

## Results

### Studies Selected

**Figure 1:**
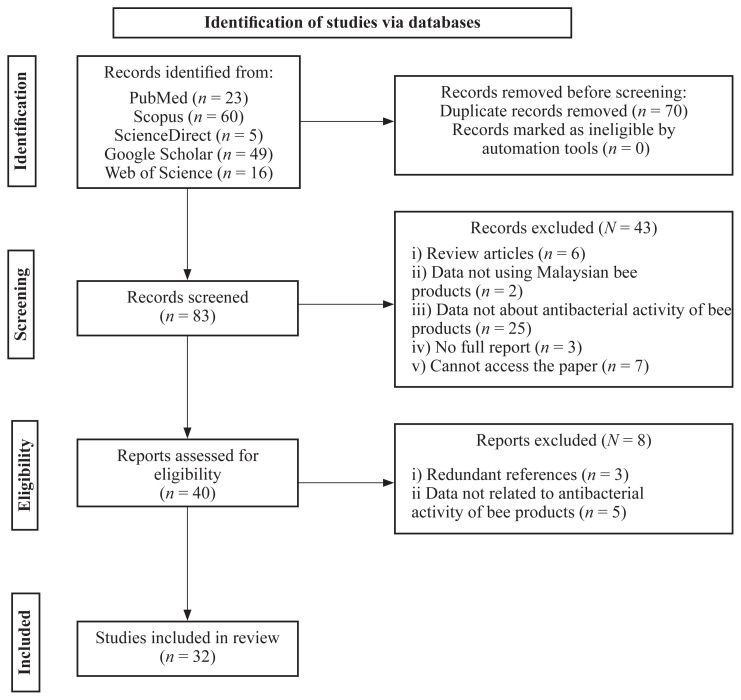
PRISMA flow diagram depicts the study selection process. In total, the search criteria were satisfied by 32 studies

All studies included in the qualitative synthesis are experimental studies. Studies included screening of antibacterial activities of any type of Malaysian bee product. A total of 153 records were obtained from five electronic databases: i) PubMed (*n* = 23); ii) Scopus (*n* = 60); iii) ScienceDirect (*n* = 5); iv) Google Scholar (*n* = 49) and v) Web of Science Core Collection (*n* = 16). After 70 duplicates were removed, 83 records were screened based on their titles and abstracts. As a result, 40 records were left for full-text thorough screening, with 8 articles excluded. Finally, a total of 32 articles were included in the study, as shown in Figure 1. Out of 32 studies included, 23 were on honey, 8 were on propolis and 1 was on bee bread.

### Types and Physicochemical Properties of Bee Products

In the selected studies, several types of honey are used, such as Tualang, Kelulut, Acacia, Gelam, Pineapple and Durian. Kelulut was the most widely tested honey with 11 studies, followed by Tualang, Gelam, Acacia and Pineapple with 8, 6, 4, and 2 studies, respectively. There was only one study that used Durian honey. However, four studies did not mention the types of honey used. Tumin et al. (11) mentioned several local brands of honey, such as Gelang, Hutan, Ee Feng Gu and Pucuk Daun, while Al-Talib et al. (12) mentioned the name of the commercial producer of honey which was Muda Liar. In addition, a study by Yap and Abu Bakar (13) used four types of honey from Sabah, which were young and old Mangrove and young and old Upper Mountain, while Yap et al. (14) tested on Sabah wild honey produced by *Apis andreniformis*, *Apis cerana*, *Apis nuluensis* and *Apis koschevnikovi* bees. For propolis, four studies used propolis from *Trigona itama* and two studies used propolis from *Trigona thoracica*. Meanwhile, one of the studies by Tuan Ismail et al. (15) mentioned the name of the bee species, which was *Apis mellifera*, and the other two studies did not state the types of propolis used. There was one study on bee bread that used *Heterotrigona itama* bee bread.

From all the selected studies, only a certain number of studies tested the physicochemical analysis of the honey. Tumin et al. (11) found that the pH of five brands of local Malaysian honey: Tualang, Gelang, Hutan, Ee Feng Gu and Pucuk Daun, was acidic with pH values of 3.55, 4.15, 3.81, 4.17 and 4.91, respectively. Furthermore, the pH of Tualang honey, Gelam honey and Acacia honey were also similar, which were 4.00, 3.64, and 3.45, respectively (16). Subsequently, Tumin et al. (11) also reported that five local honey brands possessed low moisture content ranging from 16.0% to 23.3%. This finding was in agreement with Julika et al. (17), who reported six selected samples of Kelulut honey: H1, H2, H3, H4, H5 and H6 had low moisture contents, which were 25.4%, 30.9%, 23.4%, 28.4%, 19.4% and 26.7%, respectively. Both studies showed that honey has a low percentage of moisture content. Other researchers were also interested in looking at the phytochemical compounds of honey, bee bread and propolis, which were believed to be among the contributing factors responsible for the antibacterial properties exhibited by these bee products. One study by Zakaria et al. (18) found a substantial amount of gallic acid compound in Kelulut honey with 61.17 μg/mL following a high-performance liquid chromatography (HPLC) analysis. Suleiman et al. (5) carried out a phytochemical screening on bee bread water extract (BBW), bee bread hot water extract (BBH) and bee bread ethanolic extract (BBE). The results revealed that the number of phytochemical constituents, including terpenoids, flavonoids and phenolic compounds such as gallic acid, caffeic acid, quercetin and kaempferol, was higher in BBE than BBW and BBH. Meanwhile, phytochemical screening in propolis by Ong et al. (19) has shown the presence of some flavonoids such as quercetin, luteolin, kaempferol, apigenin and pinocembrin. Notably, pinocembrin and kaempferol were present in great amounts ranging from 4 μg/mL to 5.9 μg/mL.

### Antibacterial Effects of Bee Products

#### Commonly Used Methodologies

A variety of methods have been used in the included studies to assess the antibacterial properties of these bee products, such as the disc diffusion method, agar well diffusion method, minimum inhibitory concentration (MIC) and minimum bactericidal concentration (MBC) test by broth dilution method, growth kinetics curves and time-kill curves. Some studies used disc diffusion and agar well diffusion methods to test the susceptibility of bacteria toward honey, propolis and bee bread. For both methods, the antibacterial agent diffuses into the agar medium and hinders the “growth” and proliferation of the microorganism. Eventually, the radius or diameters of inhibition growth zones are measured. Two out of 32 studies used both disc diffusion and agar well diffusion methods, 10 studies used the agar well diffusion method only, while 8 studies used the disc diffusion method only. However, this method cannot distinguish the bacteriostatic and bactericidal effects (20). Hence, the MIC test was the most commonly used method in the selected studies, with 71.88% (23 out of 32). From this MIC test, MBC can be determined since MBC is the counterpart measurement to MIC, in which MIC measures the lowest concentration of an antibacterial agent necessary to prevent the growth of bacteria. At the same time, MBC identifies the lowest concentration required to kill a specific type of bacteria (21) effectively. Nonetheless, 8 out of 23 studies that used the MIC test did not proceed to this MBC test. Other studies investigating antibiofilm activities used a scanning electron microscope (SEM) to observe morphological cell changes, membrane integrity and evidence of cell division before and after treatment with the bee products (22). A confocal laser scanning microscope (23) and a fluorescence microscope (19) are other devices that can be used to assess anti-biofilm activities. Biofilm reduction assay, biofilm prevention assay, determination of biofilm viability by total cell count and analysis of gene expression of bacteria after treatment with bee products are among the methods used to assess the antibiofilm properties (24).

#### Honey

Different studies used different types of honey for their research purposes. In general, it can be concluded from these studies that honey exhibits great potential to inhibit growth and kill bacteria. Kelulut honey is the most widely tested honey, and it was found to have significant antibacterial activities, although the different laboratories used different concentrations. Kelulut honey was found to be the most effective antibacterial agent against *P. aeruginosa*, with a MIC value of 3.75% (w/v) and MBC value of 12.5% (w/v) (8). A study by Al-Kafaween et al. (24) demonstrated that the MIC for Kelulut honey against *Staphylococcus aureus* was at 30% (w/v). Meanwhile, the MIC and MBC values for Kelulut honey against *Staphylococcus aureus*, *B. cereus*, *P. aeruginosa* and *Escherichia coli* were constant at 20% (w/v) (25). Intriguingly, Kelulut honey demonstrated a bacteriostatic effect at a very low concentration of 1.56% (w/v) against *B. subtilis* and *Staphylococcus aureus* and a bactericidal effect at a concentration of 3.125% (w/v) against both bacteria (26).

In addition, eight researchers studied the antibacterial properties of Tualang honey. These Tualang honey demonstrated a broad spectrum of activities against nine Gram-positive bacteria (*S. aureus*, *B. cereus*, *S. epidermis*, *S. pyogenes*, *E. faecalis*, *E. faecium*, coagulase-negative Staphylococci, methicillin-resistant *Staphylococcus aureus* and *S. agalactiae*) and 10 Gram-negative bacteria (*Escherichia coli*, *P. aeruginosa*, *S. enterica Serovar Typhimurium*, S*. flexneri*, *S. sonnei*, *K. pneumoniae*, *S. malthophilia*, *A. baumannii*, *P. mirabilis* and *E. cloacae*) as shown in Table 1. Tualang honey was the most highly potent against *S. enterica Serovar Typhimurium* by exhibiting the lowest MIC (3.125 mg/mL) and MBC (12.5 mg/mL) value compared to other bacteria; *Staphylococcus aureus*, *Escherichia coli*, *S. epidermis* and *P. aeruginosa* (21). Similarly, another study has also shown that Tualang honey possessed the strongest antibacterial activity against *S. enterica Serovar Typhimurium* although the concentration used to access the antibacterial activity was measured using volume/volume percentage concentration (20% (v/v)) (10).

A study by Al-Kafaween et al. (27) showed that MIC and MBC values for Tualang honey against *P. aeruginosa* were higher with 18.5% (w/v) and 25% (w/v), respectively, in comparison to the study by Zainol et al. (25) which exhibit MIC values of 12.5% (w/v) and MBC value of 20% (w/v), although the honey samples were tested on the same bacterium.

Gelam honey is a prized natural product produced by the Gelam tree, also known as *Malaleuca cajuputi* tree. It was reported that all concentrations (0.0625, 0.125, 0.25, 0.5 and 0.1) g/mL of Gelam honey had successfully exhibited antibacterial properties against Gram-positive bacterium, *S. mutans*, where 100 % inhibition rate was seen with the concentration of 1 g/mL Gelam honey followed by 99% inhibition rate with concentrations of 0.5 g/mL and 0.25 g/mL (28). On the other hand, Gelam honey can also inhibit and kill Gram-negative bacterium *Escherichia coli* at a concentration of 12.5% (w/v) and 15% (w/v), respectively (25).

The other Malaysian honey that four researchers have studied was Acacia honey. Acacia honey was recorded to inhibit the growth of several species of gastroenteritis bacteria, such as *Shigella flexneri*, *Shigella sonnei*, *P. aeruginosa* and *Staphylococcus aureus.* However, this honey was the most potent against *S. flexneri* because it needed a dilution of only 6.3% (v/v) to inhibit and kill them (29). Another study by Zainol et al. (25) and Al-Kafaween et al. (16) demonstrated that Acacia honey was effective against *Escherichia coli* by inhibiting them at a concentration of 25% (w/v) and killing them at a concentration of 50% (w/v). Out of 23 studies on honey, one study was on Durian honey. As shown in Table 1, Durian honey was tested against five Gram-positive bacteria and three Gram-negative bacteria. *K. pneumoniae* was the most susceptible to Durian honey by exhibiting the largest zone of inhibition (17.5 mm) compared to other bacteria (10).

#### Bee Bread

Out of 32 selected studies, only one study by Suleiman et. al (5) reported the antibacterial activity of *Heterotrigona itama* bee bread. Three extracts, including BBW, BBH and BBE, were used in this study. However, only BBE was used to assess the antimicrobial activity since it demonstrated the most promising antioxidant properties. BBE possessed antibacterial activity against the bacteria tested: *Escherichia coli*, *Salmonella typhi*, *Shigell*, and *K. pneumonia*. The strongest antimicrobial activity of this bee bread was against *Shigella* with MIC_50_ 1.617 μg/mL, while the weakest antimicrobial activity was in *K. pneumonia*, which exhibited MIC_50_ 1.923 μg/mL.

#### Propolis

Numerous researchers have conducted extensive investigations into the antibacterial properties of propolis. Their findings consistently indicate that propolis possesses notable bacteriostatic and bactericidal activity against certain pathogenic microorganisms. In a study by Manuharan et al. (30), ethanolic extract of *Trigona itama* propolis (EEP), diluted ethanol extract of propolis, and raw propolis were used against *Staphylococcus aureus*, *Bacillus cereus*, *Salmonella enterica* and *Escherichia coli*. The results by the author showed that EEP exhibited a higher zone of inhibition against the tested bacteria compared to diluted EEP and raw propolis, as shown in Table 1. This study concluded that antibacterial activity against pathogens increased with the increase of propolis concentration. In contrast, propolis extract has better antibacterial properties than raw propolis due to the extraction of some active compounds that contribute to its antibacterial activity. In addition, propolis has also been shown to have promising effects in treating acne vulgaris due to its great antibacterial activity. Tuan Ismail et al. (15) tested EEP and water extract (WEP) of Malaysian *Apis mellifera* propolis from the northern and southern regions of Peninsular Malaysia and the results demonstrated that EEP from the northern region showed the best antibacterial activity against *Propionibacterium acnes* with MIC values, 0.32 μg/mL, followed by EEP from the southern region, WEP from the southern region and WEP from northern region with MIC values 0.63 μg/mL, 625 μg/mL and 2,500 μg/mL, respectively. Besides, propolis can be developed into nanoparticles with chitosan since nanoparticles have recently gained attention in medicine because of their better efficacy, improved bioavailability and enhanced penetration ability (19). This chitosan-propolis nanoparticle formulation inhibited bacterial growth and biofilm formation by *Enterococcus faecalis* (19). Disruption of biofilm, including biofilm layer, became thin or discontinuous and some structural changes can be seen after treatment with propolis using a microscope. Another study by Ong et al. (23) demonstrated that treatment of *Staphylococcus epidermis* with cationic chitosan-propolis nanoparticles could decrease the viability of bacteria to 25% when observed under confocal laser scanning microscopy (CLSM). This is due to the reduction in biofilm formation and membrane damage.

## Discussion

This study systematically reviewed current in vitro studies related to the antibacterial activity of bee products from Malaysia, including honey, propolis and bee bread. Different types of honey and different types of bacteria were used in each study. Among the honey types used are Kelulut, Tualang, Gelam, Acacia, Pineapple, Durian and other honey. The most studied honey was Kelulut honey, with 11 studies. In Malaysia, honey production by honeybees like *Apis mellifera* had suffered from the *Varroa destructor* mite outbreak in 1996. Hence, the accessibility of locally sourced honey relies entirely on honey hunters who collect wild honey from stinger honeybee species, such as Tualang bees (*Apis dorsata*). Tualang bees predominantly nest in remote jungle areas and high above the ground, making it difficult to implement standard production procedures. On the other hand, Kelulut bees, also known as stingless bee (*Meliponini sp*.), does not possess stingers and build nests in existing cavities of trees, hives and buildings. This nesting behaviour enables cultivating stingless bees in intensive farms or homes in rural areas with controlled environments and implementing standard operating procedures. The empowerment of the stingless bee industry has a dual benefit: it directly improves high-quality honey production and supports crop pollination, which is crucial for maintaining biodiversity (40). Hence, this could be why most authors were interested in Kelulut honey.

Many factors contribute to the antibacterial properties of honey, such as acidity (low pH), low moisture content, the content of hydrogen peroxide (H_2_O_2_) and non-peroxide antibacterial compounds (41). From the results of physicochemical properties, the bee products are in acidic condition, and this low pH can inhibit the growth of several bacteria and other spoilage microorganisms. Honey’s acidic nature is primarily attributed to various organic acids, in which gluconic acid predominates. In addition, honey in its natural form is characterised by low moisture content, providing an undesirable environment for the survival of bacteria and other microorganisms (42). The antibacterial activity of honey is also mainly attributed to hydrogen peroxide. Hydrogen peroxide and gluconic acid are generated due to the oxidation of glucose by glucose oxidase. However, honey should be diluted to activate the enzyme glucose oxidase. Non-peroxide honey is a term that describes honey that maintains its antibacterial activities even when glucose oxidase is absent. It has been discovered that honey contains a substantial amount of phenolic compounds, which may explain its antibacterial activity. Among the phenolic compounds reported are protocatechuic acid, caffeic acid, vanillic acid, apigenin, kaempferol and pinocembrin (41, 43). Zakaria et al. (18), Suleiman et al. (5), and Ong et al. (19) have also reported the presence of phenolic compounds in honey, bee bread and propolis.

Many efforts have been made to search for alternative medicines that act as antibacterial agents against antibiotic-resistant bacteria (44). Honey is consumed as a food source, additives in numerous food preparations, and medicine by both old and new generations (45). Honey has been discovered to exhibit other antioxidant, anti-inflammatory, anticancer, antimicrobial, antiviral, antifungal and anti-diabetic properties (2). Malaysian honey is categorised based on the bee species or the nectar sources of the honey. There are two main categories of bee species: Meliponine (stingless bee, locally known as Kelulut) and Apis (*A. mellifera*, *A. dorsata* and *A. cerana*), also known as stinging bees. Honey can be further categorised as monofloral (Acacia, Gelam, Pineapple, Durian and Coconut honey) or polyfloral (Kelulut and Tualang honey) according to floral sources (46).

The antibacterial activity of different types of honey indicates that there is variation in the antibacterial capacity of honey, even when similar bee species produce it. This could be due to different geographical locations, seasonal conditions, flower sources, processing and storage conditions (35). Moreover, different bacterial species have varying susceptibilities to honey, but these differences can also exist between strains of the same bacterial species. Because of this, the findings for each strain cannot be applied to the entire bacterial species. However, the outcomes of the studies demonstrate that most of the honey can inhibit and kill the bacteria regardless of what concentrations they were using. Moreover, different laboratory studies were using different procedures to run the experiment. For example, the unit used to measure the MIC and MBC values were not uniform in some studies, with some studies using milligram per millimeter (mg/mL). In contrast, the others used percentage volume per volume (% (v/v)) and percentage weight per volume (% (w/v)). Similarly, the authors used different measurements for the zone of inhibition. Some used millimeters (mm), while others used the centimeter (cm) unit. The variation in the findings could also be due to the technical variation while experimenting, such as the amount of bacterial suspension used, the diluent used and the type of agar or broth. Thus, the antibacterial properties of different types of Malaysian honey cannot be compared easily.

In addition, the antibacterial activity of the Malaysian *Heterotrigona itama* BBE is due to its low pH. The low pH may result from the fermentation of bee pollen to bee bread, which produces lactic acid bacteria. Several phytochemical compounds, such as flavonoids and phenolic content, also contribute to bee bread’s antibacterial properties. The phytochemical screening analysis showed that BBE had high concentrations of flavonoids, saponins, resins, terpenoids, alkaloids, tannins, xanthoproteins, glycosides and phenols. Moreover, nine phenolic compounds, including caffeic acid, gallic acid, mangiferin, trans-ferulic acid, 2-hydroxycinnamic acid, trans-3-hydroxycinnamic acid, kaempferol, apigenin and quercetin have been revealed in BBE using HPLC analysis and this polyphenol has substantial antimicrobial activities (5).

Propolis contains various chemical and biological components that contribute to its antibacterial properties. The inhibition of both Gram-negative and Gram-positive bacteria by propolis was significant. Propolis is effective against many microorganisms, including bacteria, fungi and viruses. Flavonoids have been acknowledged as significant active components concerning their antibacterial properties (47). According to Manuharan et al. (30), Malaysian propolis can suppress Gram-negative bacteria, which is unusual for propolis from most other parts of the world. It is important to note that different geographical conditions might produce different components and active compounds in propolis, thus explaining its good inhibition against Gram-negative bacteria compared to Gram-positive bacteria. Overall, propolis exhibits antibacterial properties that are almost identical to or even better than those of antibiotics.

The study on the antimicrobial activity of WEP and EEP against *Propionibacterium acnes* has been done by Tuan Ismail et al. (15). EEP demonstrated better antimicrobial activity than WEP. Both lipid and water-soluble compounds in EEP likely contribute to this variation. The choice of extraction method and the specific solvent used can significantly affect the properties of propolis, as different solvents have the potential to modify the constituents of propolis, consequently impacting its effects. Moreover, the differences in the antimicrobial activity for EEP from the southern and northern regions may be due to the differences in chemical compounds. According to Ong et al. (19), *Enterococcus faecalis* is resistant to most available antibiotics. It also produces biofilms, which shield the microorganisms within and prevent antimicrobial agents from penetrating. The chitosan-propolis nanoformulation (F1) that has been chosen exhibited the best physicochemical properties and inhibited both bacterial growth and biofilm formation by *Enterococcus faecalis.* Nanoparticles can enhance penetration power and treatment efficacy due to their nano-scale particle size. Hence, it can penetrate biofilms effectively. Thus, this nanoformulation could be potentially developed as a therapeutic agent to combat bacterial biofilms. In addition, research by Ibrahim et al. (33) demonstrates that different bee species influence the chemical composition and biological activities of the honey, and this was the first report of the antibacterial properties of Malaysian propolis, specifically obtained from two species of stingless bees, *Geniotrigona thoracica*, and *Heterotrigona itama*. This provides some insight into the potential of Malaysian propolis. In general, propolis is a potent antibacterial agent, making it a promising natural remedy for various health conditions. However, more research is needed to understand its mechanisms of action and potential clinical applications fully.

## Conclusion

In conclusion, different types of honey, bee bread and propolis exhibited a broad spectrum of activities against several Gram-positive and Gram-negative bacteria. The potency of these bee products against particular bacteria suggests their potential to be used as an alternative therapeutic agent for certain medical conditions. The great physicochemical properties such as low pH, low moisture content and presence of phytochemical compounds are among the possible factors responsible for the antibacterial properties of these Malaysian bee products.

## Figures and Tables

**Table 1:** Overview characteristics of included studies

Reference	Type of bee products	Bacteria tested	Method used	Results
Tumin et al. (11)	Local brands of honey namely Tualang, Hutan, Gelang, Pucuk Daun, Ee Feng Gu	*Escherichia coli, Salmonella typhi, Shigella sonnei, Pseudomonas aeruginosa, Staphylococcus aureus*	Disc diffusion method, Agar well diffusion method, MIC	Zone of inhibition against all tested bacteria (range) Pucuk Daun: 20 mm–30 mm Tualang: 15 mm–36 mm Ee Feng Gu: 15 mm–35 mm Gelang and Hutan had no zone of inhibition. MIC value range against tested bacteria Tualang: 48.75 μg/mL–195 μg/mL Gelang: 17.5 μg/mL–140 μg/mL Hutan: 28.75 μg/mL–230 μg/mL Ee Feng Gu: 135 μg/mL–270 μg/mL Pucuk Daun: 5 μg/mL– 160 μg/mL
Tan et al. (31)	Tualang honey	*Streptococcus pyogenes, coagulase-negative Staphylococci, MRSA, Streptococcus agalactiae, Stenotrophomonas maltophilia, Acinetobacter baumannii, Salmonella enterica Serovar typhi, Pseudomonas aeruginosa, Proteus mirabilis, Shigella flexneri, Escherichia coli, Enterobacter cloacae*	MIC, MBC	MIC value range against tested bacteria 8.75% (w/v)–25% (w/v) MBC value range against tested bacteria 20% (w/v)– >25% (w/v)
Al-Talib et al. (12)	Honey from local commercial producers (Muda Liar)	*Staphylococcus aureus, methicillin-resistant Staphylococcus aureus (MRSA), Streptococcus bovis, Streptococcus pyogenes, Enterococcus faecalis, Listeria monocytogenes, Pseudomonas aeruginosa, Escherichia coli, Acinetobacter baumannii, Klebsiella pneumonia, Shigella sonnei*	Broth dilution method	*Streptococcus pyogenes* was the most sensitive - complete inhibition at 30% broth dilution *Staphylococcus aureus* and *Enterococcus faecalis* were the most resistant - complete inhibition at 80% broth dilution IC_50_ Ranged from 4% (*Streptococcus bovis* and *Pseudomonas aeruginosa*)–15% (MRSA)
Sayadi et al. (29)	Gelam, Pineapple, Tualang, Acacia honey	*Shigella flexneri Shigella sonnei Pseudomonas aeruginosa, Staphylococcus aeureus*	MIC, MBC	MIC value range against tested bacteria Gelam: 6.3%–25% (v/v) Tualang: 6.3%–25% (v/v) Pineapple: 12.5%–25% (v/v) Acacia: 6.3%–12.5% (v/v) MBCs value range against tested bacteria Gelam: 25%–>25% (v/v) Tualang: 6.3%–>25% (v/v) Pineapple: 12.5%–>25% (v/v) Acacia: 12.5%–>25% (v/v)
Zainol et al. (25)	Acacia, Gelam, Pineapple, Kelulut, Tualang honey	*Staphylococcus aureus, Bacillus cereus, Escherichia coli, Pseudomonas aeruginosa*	MIC, MBC	MIC value range against tested bacteria Acacia: 15%–25 % (w/v) Gelam: 5%–15 % (w/v) Kelulut: 20% (w/v) Pineapple: 15%–25 % (w/v) Tualang: 10%–20 % (w/v) MBCs value range against tested bacteria Acacia: 25%–50% (w/v) Gelam: 6.25%–15% (w/v) Kelulut: 20% (w/v) Pineapple: 25%–50% (w/v) Tualang: 15%–25% (w/v)
Ng et al. (28)	Gelam honey	*Streptococcus mutans*	Disc diffusion method	Gelam honey with 1 g/mL concentration was able to inhibit bacterial growth completely (100%)
Ng et al. (10)	Tualang, Gelam, Durian	*Staphylococcus aureus, Staphylococcus epidermidis, Enterococcus faecium, Enterococcus faecalis, Escherichia coli, Salmonella enterica serovar Typhimurium, Klebsiella pneumoniae*	Agar well diffusion method, MIC, MBC	MIC value range against tested bacteria Tualang 125 mg/mL–500 mg/mL Gelam 125 mg/mL–1,000 mg/mL Durian 125 mg/mL–1,000 mg/mL MBCs value range against tested bacteria Tualang: 500 mg/mL–2,000 mg/mL Gelam: 125 mg/mL–2,000 mg/mL Durian: N/A–2,000 mg/mL Zone of inhibition against tested bacteria (range) Tualang: < 35 mm Gelam: < 30 mm Durian: < 20 mm
Yap and Abu Bakar (13)	Old upper mountain, Old mangrove, Young mangrove, Young upper mountain honey	*Staphylococcus aureus, Bacillus cereus, Bacillus subtilis, Escherichia coli, Salmonella enteritidis*	Disc diffusion method	Zone of inhibition against tested bacteria (range) Old upper mountain 1.00 mm–6.00 mm Old mangrove 1.00 mm–5.00 mm Young mangrove 1.00 mm–4.00 mm Young upper mountain 1.00 mm–5.00 mm
Ng and Lim (32)	Gelam honey	*Staphylococcus aureus methicillin sensitive* (MSSA) *strains* (ATCC 25923 and ATCC 6538) and methicillin-resistant (MRSA) strains (ATCC 33592 and ATCC 33591)	Agar well diffusion method	Zone of inhibition against tested bacteria MSSA ATCC 25923: 0.0–10.7 mm MSSA ATCC 6538: 0.0–10.0 mm MRSA ATCC 33592: 0.0–13.0 mm MRSA ATCC 33591: 0.0–13.7 mm
Shehu et al. (21)	Tualang honey	*Staphylococcus aureus, Staphylococcus epidermidis, Escherichia coli, Pseudomonas aeruginosa, S. enterica Serovar Typhimurium*	MIC, MBC	MIC value range against tested bacteria 3.125 mg/mL–25 mg/mL MBCs value range against tested bacteria 12.5 mg/mL–50 mg/mL
Ibrahim et al. (33)	*Heterotrigona itama* (MHI), *Geniotrigona thoracica* (MGT) propolis	*Staphylococcus aureus, Bacillus subtilis, Enterococcus faecalis, Listeria monocytogen, Acinetobacter baumannii, Salmonella typhi, Escherichia coli*	Disc diffusion method, MIC	Zone of inhibition against tested bacteria (range) MHI 6 mm–14 mm MGT 6 mm–7 mm MIC value range against tested bacteria MHI 5 mg/mL–10 mg/mL MGT 20 mg/mL–> 40 mg/mL
Yap et al. (14)	Sabah wild honey: *Apis cerana, Apis andreniformis, Apis nuluensis, Apis koschevnikovi*	*Staphylococcus aureus, Bacillus cereus, Bacillus subtilis, Escherichia coli, Salmonella enteritidis*	Disc diffusion method, MIC	Zone of inhibition for both 80% and absolute methanol extract against tested bacteria (range) *Apis cerana* 1 mm–16 mm *Apis andreniformis* N/A–5 mm *Apis nuluensis* 1 mm–7 mm *Apis koschevnikovi* N/A–5 mm The MICs value range for both 80% and absolute methanol extract against tested bacteria *Apis cerana* 31.25 L/mL–250 L/mL *Apis andreniformis* 62.5 L/mL–250 L/mL *Apis nuluensis* 31.25 L/mL–250 L/mL *Apis koschevnikovi* 125 L/mL–250 L/mL
Ong et al. (19)	Chitosan-propolis nanoparticle	*Enterococcus faecalis*	Biofilm assay, visualisation of biofilm using -SEM -fluorescent microscope	Percentage of survival of biofilm bacteria when treated with chitosan-propolis nanoparticle 100 μg/mL - 30% survival of biofilm 300 μg/mL - 10% survival of biofilm Visualisation of biofilm (SEM) Disruption in biofilm and inhibition of biofilm can be seen by some irregular patches of clear areas. Visualisation of biofilm (fluorescent microscope) Discontinuous and thin biofilm layer
Tuksitha et al. (34)	Kelulut honey with three different species; *Geniotrigona thoracica, Heterotrigona itama, Heterotrigona erythrogastra*	*Staphylococcus aureus, Staphylococcus intermedius B, Staphylococcus xylosus, Streptococcus alactolyticus, Citrobacter koseri, Escherichia coli, Klebsiella pneumonia, Pseudomonas aeruginosa, Salmonella cholerasuis, Vibrio parahaemolyticus*	Disc diffusion method, MIC, MBC	Zone of inhibition of honey against tested bacteria (range) *Geniotrigona thoracica* 1.23 cm–3.00 cm *Heterotrigona itama* N/A–3.30 cm *Heterotrigona erythrogastra* 1.00 cm–3.37 cm MIC value range against tested bacteria *Geniotrigona thoracica* 3% (w/w)–5% (w/w) *Heterotrigona itama* 5% (w/w)–10% (w/w) *Heterotrigona erythrogastra* 2% (w/w)–5% (w/w) MBCs value range against tested bacteria *Geniotrigona thoracica* 5% (w/w)–10% (w/w) *Heterotrigona itama* 10% (w/w)–20% (w/w) *Heterotrigona erythrogastra* 10% (w/w)–15% (w/w)
Julika et al. (17)	Kelulut honey with six different samples: H1, H2, H3, H4, H5 and H6	*Escherichia coli, Bacillus sp*	Disc diffusion method	Zone of inhibition against tested bacteria *Escherichia coli* - 11 mm *Bacillus sp*. - 10 mm–24 mm
Mohd Aspar et al. (8)	Kelulut, Tualang and Acacia honey	*Staphylococcus aereus, Streptococcus pyogenes, Enterococcus faecalis, Escherichia coli, Pseudomonas aeruginosa, Salmonella enterica serovar Typhimurium, Proteus mirabilis, Klebsiella pneumoniae*	MIC, MBC	MIC value range against tested bacteria Kelulut: 3.75% (w/v)–20% (w/v) Tualang: 20% (w/v)–40% (w/v) Acacia: 30% (w/v)–50% (w/v) MBCs value range against tested bacteria Kelulut 12.5% (w/v)–50% (w/v) Tualang 40% (w/v)–>90% (w/v) Acacia 50% (w/v)–>90% (w/v)
Omar et al. (35)	Kelulut honey with three different species: Multifloral honey from *G. thoracica* (GTM), Unifloral honey from *Geniotrigona thoracica* (Senduduk), Multifloral honey from *Heterotrigona itama* (HTM)	MRSA, *Pseudomonas aeruginosa, Klebsilla pneumonia, Streptococcus pyogenes, Staphylococcus aureus, Escherichia coli*	Agar well diffusion method, MIC, MBC	Zone of inhibition against tested bacteria (range) GTM: 9.7 mm–24.3 mm Senduduk: 7.7 mm–21.3 mm HTM: 13.6 mm (only effective against Staphylococcus aereus) MIC value range against tested bacteria GTM: 3.13% (v/v) Senduduk: 3.13 % (v/v)–6.25% (v/v) HTM: 6.25 % (v/v)–12.5% (v/v) MBCs value range against tested bacteria GTM: 6.25% (v/v)–12.5% (v/v) Senduduk: 25% (v/v) HTM: 25% (v/v)
Ong et al. (23)	Chitosan-propolis nanoparticle	*Staphylococcus epidermidis*	Confocal laser scanning microscopy, Biofilm imaging (SEM)	Confocal scanning laser microscopy The viability of bacteria has been reduced to 25% The membrane has been disrupted Biofilm formation has been reduced Biofilm imaging by SEM The bacterial count has been decreased, and biofilm has been disrupted
Wan Yusop et al. (36)	*Trigona itama* propolis	*Escherichia coli, Staphylococcus aureus*	Disk diffusion method	Zone of inhibition (range) S. aureus 4 mm–10 mm *Escherichia* coli 4 mm–10 mm
Ismail et al. (37)	Geopropolis from *Heterotrigona itama* bee	*Enterococcus faecalis*	MIC, MBC, Agar well diffusion method	Zone of inhibition mean: 6.21 mm MIC 8 mg/mL MBC 16 mg/mL
Al-Kafaween et al. (27)	Tualang honey	*Pseudomonas aeruginosa, Streptococcus pyogenes*	MIC, MBC, SEM	MICs *Pseudomonas aeruginosa* 18.5% (w/v) *Streptococcus pyogenes* 13% (w/v) MBCs *Pseudomonas aeruginosa* 25% (w/v) *Streptococcus pyogenes* 25% (w/v) SEM Honey-treated cells have appeared to be shortened and to have distorted shapes Clumping and cell aggregation have been increased
Al-Kafaween et al. (22)	Kelulut honey	*Pseudomonas aeruginosa, Streptococcus pyogenes*	MIC, MBC, Agar well diffusion method	Zones of inhibitions *Pseudomonas aeruginosa* 25.2 mm S*treptococcus pyogenes* 26.7 mm MICs value against tested bacteria 20% (w/v) MBCs value against tested bacteria 25% (w/v)
Syed Yaacob et al. (26	Kelulut honey Four samples namely Sy-1, Sy-2, Sy-3, Sy-4	*Bacillus subtilis, Staphylococcus aereus, Escherichia coli, Pseudomonas aeruginosa*	Agar well diffusion method, MIC, MBC	Mean zone of inhibition against tested bacteria (range) Sy-1: 8 mm–27 mm Sy-2: 4 mm–23 mm Sy-3: 5 mm–27 mm Sy-4: 6 mm–23 mm MICs value against tested bacteria Sy-1: 1.56% (w/v)–3.125% (w/v) Sy-2: 6.25% (w/v) Sy-3: 3.125% (w/v)–6.25% (w/v) Sy-4: 3.125% (w/v)–6.25% (w/v) MBCs value against tested bacteria Sy-1: 3.125% (w/v)–6.25% (w/v) Sy-2: 6.25% (w/v) Sy-3: 3.125% (w/v)–6.25% (w/v) Sy-4: 3.125% (w/v)–6.25% (w/v)
Zakaria et al. (18)	Kelulut honey Sample 1, Sample 2, Sample 3	*Staphylococcus aureus, Escherichia coli*	Disc diffusion method	Zone of inhibition against tested bacteria (range) Honey Sample 1: 8.00 mm–15.17 mm Honey Sample 2: 12.67 mm–22.67 mm Honey Sample 3: 12.33 mm–22.67 mm
Al-Kafaween et al. (24)	Kelulut honey	*Staphylococcus aureus, Escherichia coli, Pseudomonas aeruginosa*	Biofilm reduction assay, MIC	Biofilm reduction assay The most effective concentration to reduce the biofilm mass Staphylococcus aureus (39%), *Escherichia coli* (37%), and *Pseudomonas aeruginosa* (41%) was 40% (w/v) MICs value against tested bacteria 30% (w/v)
Al-Kafaween et al. (38)	Kelulut honey	*Streptococcus pneumoniae*	Agar well diffusion method, MIC, MBC	Zones of inhibition (mm) 10% honey concentration: 0 25% honey concentration: 9.3 50% honey concentration: 16.6 75% honey concentration: 19.1 100% honey concentration: 22.2 MIC value 25% (w/v) MBCs value 30% (w/v)
Al-Kafaween et al. (16)	Tualang, Gelam, Acacia honey	*Escherichia coli*	MIC, MBC	MICs value Tualang 20% (w/v) Gelam 20% (w/v) Acacia 25% (w/v) MBCs value Tualang 25% (w/v) Gelam 25% (w/v) Acacia 50% (w/v)
Manuharan et al. (30)	*Trigona itama* propolis	*Staphylococcus aureus, Bacillus subtilis, Escherichia coli, Salmonella enterika*	Agar well diffusion method	Inhibition radius against tested bacteria EEP: 11 mm–25 mm Diluted EEP: 1 mm–10 mm Raw propolis: N/A–8 mm
Shalsh et al. (9)	Kelulut honey brand 1 (K1), brand 2 (K2)	*Escherichia coli, Salmonella typhimurium, Staphylococcus aureus, Streptococcus pyogenes*	Disc diffusion method, Agar well diffusion method, MIC, MBC	Zone of inhibition at 80% concentration against tested bacteria (disc diffusion assay) K1: 2 mm K2: 1.5 mm K1 and K2 at 80% concentration only effective against *Streptococcus pyogenes* Zone of inhibition (range) at 100% concentration against tested bacteria (disc diffusion assay) K1: 2 mm–4 mm K2: 2 mm–3.3 mm Zone of inhibition at 80% concentration against tested bacteria (agar well diffusion assay) K1: 2.3 mm–3 mm K2: 2 mm–2.4 mm K1 and K2 at 80% concentration only effective against *Staphylococcus aureus* and *Streptococcus pyogenes* Zone of inhibition at 100% concentration (agar well diffusion assay) K1: 2.4 mm–9.3 mm K2: 1.6 mm–8.2 mm MIC value range against tested bacteria 8.5 mg/mL–70 mg/mL
Suleiman et al. (5)	*Heterotrigona itama* bee bread	*Klebsilla pneumonia, Escherichia coli, Shigella, Salmonella typhi*	MIC	MIC_50_ *Shigella* (1.617 μg/mL), *Salmonella typhi* (1.813 μg/mL) *Escherichia coli* (1.914 μg/mL) *Klebsilla pneumonia* (1.923 μg/mL)
Tuan Ismail et al. (39)	*Trigona thoracica* propolis	*Streptococcus mutans, Streptococcus sobrinus*	Agar well diffusion method, MIC	Zone of inhibition of propolis against tested bacteria *Streptococcus mutans* (14 mm) *Streptococcus sobrinus* (18 mm) MICs value against tested bacteria *Streptococcus mutans* (625 μg/mL) *Streptococcus sobrinus* (625 μg/mL)
Tuan Ismail et al. (15)	*Apis mellifera* propolis: WEP and EEP	*Propionibacterium acnes*	Agar well diffusion method, MIC	Zone of inhibition EEP from the southern region (16 mm) EEP from the northern region (29 mm) WEP from the southern region (26 mm) WEP from the northern region (24 mm) MICs value EEP from the southern region (0.63 μg/mL) EEP from the northern region (0.32 μg/mL) WEP from the southern region (625 μg/mL) WEP from the northern region (2500 μg/mL)

## Appendix

### PubMed

(((((((honey[Title/Abstract]) OR (propolis[Title/Abstract])) OR (“royal jelly”[Title/Abstract])) OR (“bee wax”[Title/Abstract])) OR (“bee venom”[Title/Abstract])) OR (“bee bread”[Title/Abstract])) AND ((Malaysia[Title/Abstract]) OR (Malaysian[Title/Abstract]))) AND (((antibacteria[Title/Abstract]) OR (antibacterial[Title/Abstract])) OR (antimicrobial[Title/Abstract]))

### Scopus

TITLE-ABS(Honey OR “royal jelly” OR propolis OR “bee wax” OR “bee venom” OR “bee bread”) AND TITLE-ABS(Malaysia OR Malaysian) AND TITLE-ABS(Antibacterial OR antibacteria OR antimicrobial)

### ScienceDirect

(Honey OR “royal jelly” OR propolis OR “bee wax” OR “bee venom” OR “bee bread”) (Malaysia OR Malaysian) (Antibacterial OR antibacteria OR antimicrobial)

### Google Scholar

allintitle:( Honey OR “royal jelly” OR propolis OR “bee wax” OR “bee venom” OR “bee bread”) (Malaysia OR Malaysian) (Antibacterial OR antibacteria OR antimicrobial)

### Web of Science Core Collection

(TI= Honey OR “royal jelly” OR propolis OR “bee wax” OR “bee venom” OR “bee bread”) AND (TI=Malaysia OR TI=Malaysian) AND (TI=Antibacterial OR TI=antibacteria OR TI=antimicrobial)
